# The Role of Bromine in Therapeutics and Bromal Hydrate

**Published:** 1894-07-14

**Authors:** Benjamin Ward Richardson


### The Role of Bromine in Therapeutics and Bromal Hydrate.

By Sir Benjamin Ward Richardson, M.D., F.R.S.

The part played by bromine in therapeutics is now very extensive, and from some points of view unique. I remember a time when bromine and bromides played no part in medicine, and when bromine itself belonged exclusively to the domain of the chemists. It was discovered about the year 1826 by Balard of Montpelier, and was originally called muride. Afterwards from its peculiar penetrating odour it was called brome, then bromine, from the Greek bromos. It was extracted originally from marine plants, and was found in the ashes of seaweeds, together with iodine. Balard found it also in the ashes of the Janthina violacea, a testaceous mollusc. It was also found as a part of the saline portion of the sea, and was obtained from the substance left after chloride of sodium had been removed from sea water by crystallisation, in the residue called bittern, and this latter indeed became its chief source.

A theory was at one time started that bromine, iodine, chlorine, and oxygen are one substance presenting different conditions, probably, as we should now say, by the molecules of the element combining with each other, as oxygen combines in the substance called ozone. The late Professor Wilson was, if I remember rightly, the originator of this theory, and said a great deal in support of it. Like the three elements, chlorine, oxygen, and iodine, it is a negative electric, and in its combining proportions bromine presents features which would seem to connect it with the family of substances named. Like all these substances, too, it acts with great intensity on dead organic matter, decomposing it much in the same way as ozone does, and indeed its use as a destroyer of infectious matter has been very loudly and properly declared by many experimenters who have employed it for this purpose. Its pungency, however, leads it to be less manageable than iodine in the sick room, and I do not know that it is more efficient.

In my researches on the action of different elements on animal bodies I followed out a long series of experiments with bromine, using it in combination with water, alcohol, ether, and amyl hydride, in all of which it is more or less soluble. In its concentrated form the vapour of bromine is an excessive irritant, when inhaled. This was discovered very early by those chemists who studied its properties, and as they found that so small a quantity as one minim infused by the breath into birds and small animals caused rapid disturbance and sometimes death, they became afraid of its toxic properties, a fear which I doubt not interrupted for some years its introduction into medicine. In my work I proceeded very carefully in administration, allowing it to be inhaled from ethereal solutions of different strengths. The conclusion I came to was that while in large doses it acted as an irritant, in smaller doses it produced paralysis of the vascular mechanism of secreting glands by which they were made to throw out an abnormal amount of secreted fluid. I found also that it produced a certain narcotic effect, not a true anæsthesia, but a nervous inactivity, which by continued administration led to phenomena of muscular paresis, approaching somewhat in effect those produced by amyl nitrite. The inquiry led me to introduce into use the various organic bromides which are now so largely employed; bromides of quinine, iron, and strychnine especially.

It may be worth while to consider or reconsider the value of several of the bromine medicines, but on this occasion I am anxious to direct attention to one that has been much neglected; namely bromal hydrate. Bromal hydrate is the analogue in the bromine series of the chloral hydrate in the chlorine series. In 1870 I used this substance both in experiment and practice, as did also Dr. Steinman, of Berlin, and Dr. John Dougall, of Glasgow; and their results, independently carried out, tallied most satisfactorily with my own. We were of one mind in thinking that this hydrate would in time prove a good addition to therapeutics.

My first observations led me rather to depreciate the value of bromal hydrate. The dose of it required to produce specific symptoms was much smaller than that required for the corresponding chloral compound, five grains of the bromal hydrate being equal to ten of the chloral hydrate. The symptoms that followed its administration were a kind of narcotism attended with muscular prostration but with less insensibility than follows from chloral hydrate. It was possible with it to produce really deep anæsthesia, but this was only brought about when the dose was unusually large, and then there followed such sudden and extreme decrease of animal temperature that signs of asphyxia from condensation of water in the bronchial passages—hydrops bronchialis—were developed with fatal consequences to one or two of the inferior animals subjected to its influences. This effect was so striking that I was led to think of the use of bromal hydrate as a good remedy for the reduction of temperature in high febrile states; and if I have for convenience sake been brought of late to use chloral hydrate preferentially for the same condition, it is only as a matter of convenience, the bromal hydrate being rather difficult to procure in the pure state.

When it is obtained pure, bromal hydrate is a crystalline substance not unlike chloral hydrate, and can be administered much in the same manner, but, as I have said, in a smaller dose. Three to five grains of it dissolved in an ounce of water form a sufficient dose for anti-febrile purposes. It can be combined with all substances that are compatible with chloral hydrate. It can be given with chloroform water, and half a drachm of glycerine added to each dose goes well. It can also be prescribed in combination with liquor ammoniæ acetatis.

A point of great interest connected with the physiological action of bromal hydrate is seen in comparing its action with that of chloral hydrate. It illustrates how a difference of chemical elementary constitution and of weight modifies physiological action; how the heavier bromine in combination differs in action from chlorine in similar combination, and from the similar compounds of the heavier iodine. The science of therapeutics, as an exact science, and therefore more practical than now exists, will ultimately rest on a full knowledge of these distinctions.

